# Catalytic Properties of the Isolated Diaphorase Fragment of the NAD^+^-Reducing [NiFe]-Hydrogenase from *Ralstonia eutropha*

**DOI:** 10.1371/journal.pone.0025939

**Published:** 2011-10-10

**Authors:** Lars Lauterbach, Zulkifli Idris, Kylie A. Vincent, Oliver Lenz

**Affiliations:** 1 Institute of Biology, Department of Microbiology, Humboldt-Universität zu Berlin, Berlin, Germany; 2 Department of Chemistry, Inorganic Chemistry Laboratory, University of Oxford, Oxford, United Kingdom; Auburn University, United States of America

## Abstract

The NAD^+^-reducing soluble hydrogenase (SH) from *Ralstonia eutropha* H16 catalyzes the H_2_-driven reduction of NAD^+^, as well as reverse electron transfer from NADH to H^+^, in the presence of O_2_. It comprises six subunits, HoxHYFUI_2_, and incorporates a [NiFe] H^+^/H_2_ cycling catalytic centre, two non-covalently bound flavin mononucleotide (FMN) groups and an iron-sulfur cluster relay for electron transfer. This study provides the first characterization of the diaphorase sub-complex made up of HoxF and HoxU. Sequence comparisons with the closely related peripheral subunits of Complex I in combination with UV/Vis spectroscopy and the quantification of the metal and FMN content revealed that HoxFU accommodates a [2Fe2S] cluster, FMN and a series of [4Fe4S] clusters. Protein film electrochemistry (PFE) experiments show clear electrocatalytic activity for both NAD^+^ reduction and NADH oxidation with minimal overpotential relative to the potential of the NAD^+^/NADH couple. Michaelis-Menten constants of 56 µM and 197 µM were determined for NADH and NAD^+^, respectively. Catalysis in both directions is product inhibited with *K*_I_ values of around 0.2 mM. In PFE experiments, the electrocatalytic current was unaffected by O_2_, however in aerobic solution assays, a moderate superoxide production rate of 54 nmol per mg of protein was observed, meaning that the formation of reactive oxygen species (ROS) observed for the native SH can be attributed mainly to HoxFU. The results are discussed in terms of their implications for aerobic functioning of the SH and possible control mechanism for the direction of catalysis.

## Introduction

One obvious strategy of evolution is the recurrent use of conserved protein domains or even whole catalytic modules in different contexts allowing the connection of previously unlinked catalytic functions. One prominent example is Complex I, which usually connects the oxidation of NADH with the reduction of ubiquinone coupled to transmembrane H^+^ pumping, thereby establishing a proton-motive force. Substructures of Complex I are found as constituents of several different proteins and protein complexes in all three domains of life [1]. For instance, the NADH dehydrogenase/diaphorase module of Complex I is part of NAD^+^-reducing formate dehydrogenases, and hydrogenases [2], [3] (see Figs. S1,S2,S3). Here we concentrate on the functional characterization of the NADH dehydrogenase module of the NAD^+^-reducing soluble [NiFe]-hydrogenase (SH) from the Knallgas bacterium *Ralstonia eutropha* H16 [4], [5], [6], [7], [8]. The SH belongs to a subclass of “bidirectional” [NiFe]-hydrogenases and provides cells with reducing equivalents in the form of NADH generated from H_2_ oxidation. The thermodynamic potentials for the 2H^+^/H_2_ couple (−410 mV) and the NAD^+^/NADH couple (−320 mV) at pH 7.0 are closely spaced, so the catalytic direction is susceptible to small changes in reactant/product concentrations. The SH may also function as an electron valve *in vivo* under conditions of excessive reductant supply, coupling NADH oxidation to H^+^ reduction [9], as proposed for the cyanobacterial bidirectional hydrogenases [10]. Catalytic activity of *R. eutropha* SH is sustained in both directions in the presence of O_2_ [7] which makes the enzyme attractive for biotechnological applications. Thus the SH belongs to a group of ‘O_2_ tolerant’ [NiFe]-hydrogenases including the membrane-bound hydrogenase from *R. eutropha* and the Hyd 1 enzymes of *Aquifex aeolicus*, and *Escherichia coli* [11], [12], [13].

**Figure 1:**
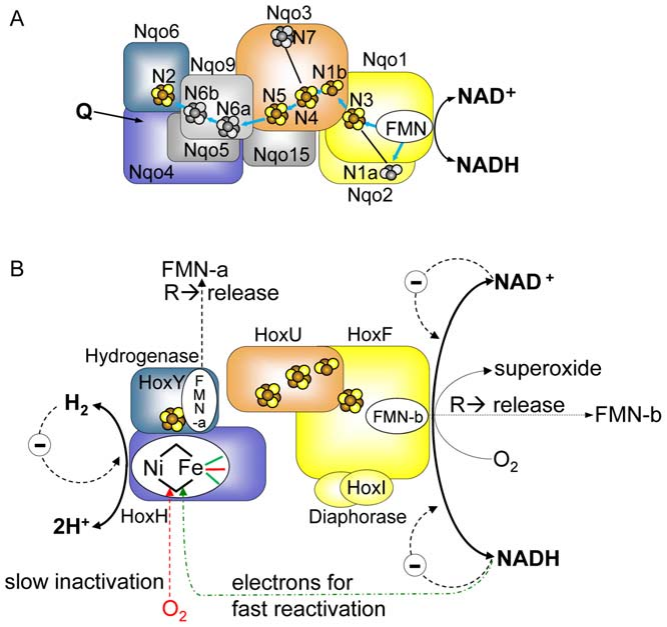
Modular structure and proposed cofactor arrangement and function of the soluble NAD^+^-reducing [NiFe]-hydrogenase of *R. eutropha* based on the results of the present study and references [**7]**, [15], [16], [21], [51****]. Panel A displays the cofactors of the hydrophilic part of *T. thermophilus* Complex I [14]. Orthologous subunits of Complex I and SH carry the same colours. Iron-sulfur clusters, which are conserved in the SH, are colored in yellow/brown, those which are not present in the SH are shown in grey. The proposed electron transfer chain in Complex I is indicated by blue arrows. The N2 cluster localized in the Nqo6 subunit of Complex I corresponds to the iron-sulfur cluster in the hydrogenase small subunit HoxY (see Figs. S1,S2,S3). The proposed quinone-binding site (Q), which is situated in Nqo4, is indicated by an arrow [17]. Panel B shows the current SH model, the proposed localization of the individual cofactors and reactions taking place at the SH. The CN^−^ ligands and the CO ligand of the Ni-Fe active site iron are shown in green and red, respectively. For sake of clarity, the hydrogenase and diaphorase modules are drawn separately. Main physiological reactions are shown in bold lines and fonts. “R” stands for “under reducing conditions”, “Θ” for “product inhibition”. Dashed lines represent effects, but without information of effect location. NADH-derived electrons for fast reactivation of the oxidized active site are passed through the FMN cofactors and the FeS clusters. For details see text and references [7], [15], [16], [21], [51].

*R. eutropha* SH comprises six subunits, HoxHYFUI_2_ and can be considered as two catalytic moieties, HoxHY and HoxFU which harbour the hydrogen cycling and diaphorase (NAD^+^/NADH cycling) activities respectively, Figure 1. All subunits of the SH, except for HoxI, have orthologous counterparts within the peripheral arm of Complex I. HoxH and HoxY share similarities with the Nqo4 and Nqo6 subunits (nomenclature according to the crystallized peripheral arm of Complex I from *Thermus thermophilus* [14]), whereby the quinone-binding site in Nqo4 is replaced by the Ni-Fe active site in HoxH [14]. The SH diaphorase module is similar to parts of the Nqo1, Nqo2, and Nqo3 subunits (Figure 1). HoxF resembles a fusion protein of Nqo1 and Nqo2 (Figs. S1,S2), lacking the [2Fe-2S] cluster N1a, while HoxU resembles a truncated version of Nqo3 that has lost its C-terminal portion including the remote cubane N7 (Figure 1 and Figure S3). The reversible transfer of two electrons from H_2_ oxidation at the Ni-Fe active site in HoxH to the FMN catalytic centre of HoxF is mediated by an electron relay chain of iron-sulphur clusters. The role of an additional flavin mononucleotide (FMN) residing in HoxY remains uncertain [4], [5], [6], [15], [16].

Unlike the *Rhodococcus opacus* SH, which *in vitro* easily dissociates into the two different moieties [17], [18], the heterotetrameric structure of the *R. eutropha* SH remains stable [19]. Only HoxI readily separates from the HoxHYFU core, under high ionic strength and alkaline pH [6].

The one-electron transfer capability of the flavin cofactors in the presence of O_2_ has the drawback of the potential production of reactive oxygen species (ROS), which can have major effects on cellular physiology. Indeed, considerable ROS production has been observed both for SH and Complex I [20], [21]. In the case of Complex I, it has been postulated that the [2Fe-2S] cluster N1a in Nqo2, which lies outside the main electron transfer chain (Figure 1B), diminishes the production of reactive oxygen species (ROS) by accepting electrons from the reduced flavin [22], [23]. Notably, a [2Fe-2S] cluster at the corresponding position is absent in the SH.

We have used genetic strategies to separate and isolate, individually, the HoxHY and HoxFU moieties. The isolated HoxHY module shows H_2_ oxidation and H^+^ reduction activity in solution assays and in protein film electrochemistry (PFE) experiments [16]. We have now applied a similar strategy to isolate and characterize the HoxFU diaphorase moiety independently, involving overproduction of the heterodimeric HoxFU protein in *R. eutropha* and purification of the protein to homogeneity. Biochemical, electrochemical and spectroscopic methods were used to probe details of catalytic bias, ROS production and O_2_ tolerance. Results are interpreted alongside the behavior and physiological role of the native SH (HoxHYFUI_2_) and the characteristics reported for Complex I.

## Results

### Homologous overproduction and purification of the functional SH diaphorase moiety

For homologous overproduction and subsequent purification of HoxFU, a plasmid was used that originally contained the SH-related *hoxFUYHWI* operon. From this plasmid, the *hoxYH* genes, encoding the hydrogenase module, were deleted and the 5′ end of *hoxF* was equipped with a *Strep*-tagII-encoding sequence.

In order to test whether the resulting plasmid pGE553 encodes functional *hoxFU*, it was conjugatively transferred to the *R. eutropha* derivative HF903, which carries an in-frame deletion in the *hoxFU* genes. Table 1 shows data confirming that plasmid pGE553 indeed restores H_2_-dependent NAD^+^ reducing activity. The observation that only 50% of the wild-type activity was obtained can be explained by the fact that the *in trans* complementation provokes a spatial separation of the synthesis of the hydrogenase and diaphorase moieties, which may lead to a less efficient assembly of the modules *in vivo*.

**Table 1:** SH activities in CTAB-treated cells of different *R. eutropha* derivatives grown in FGN medium.

		H_2_→NAD^+^ activity
*R. eutropha* strain	Relevant characteristics	[Units mg^−1^]
HF798	SH^+^	5.7±1.0
HF903	SH^−^ (Δ*hoxFU*)	<0.001
HF903 (pGE553)	SH^+^, p*hox_Strep_FU*	2.8±0.2

Values represent the arithmetic mean and standard deviation of three independent experiments.

**Figure 2:**
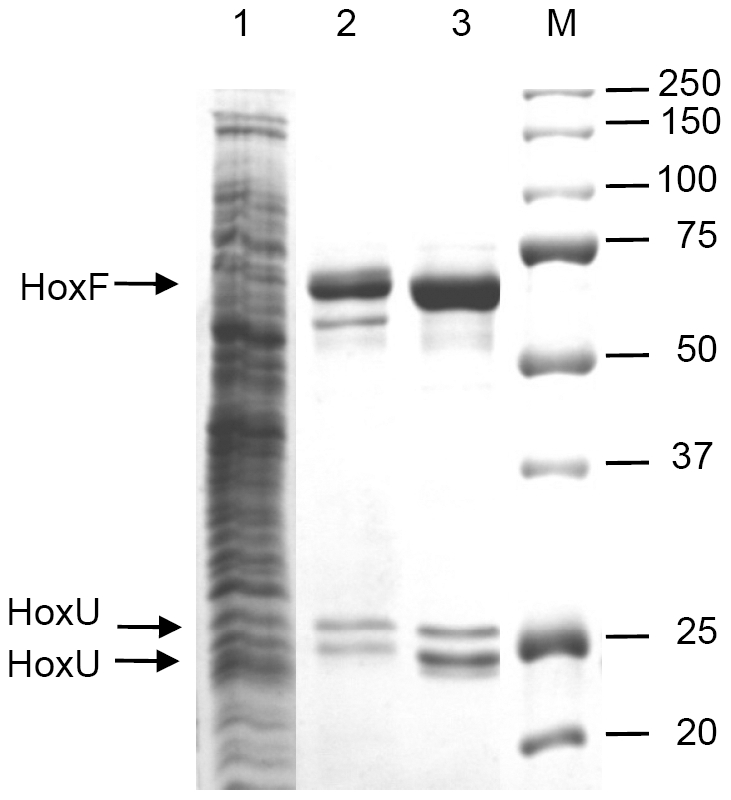
Purification of the HoxFU module of the *R. eutropha* SH. Soluble extract (20 µg, lane 1) and purified protein after *Strep*-Tactin affinity chromatography (3 µg, lane 2) and subsequent size exclusion chromatography (3 µg, lane 3) were separated by SDS-PAGE and stained with Coomassie blue. A standard protein ladder and the corresponding sizes in kDa are shown in lane M. Arrows indicate the HoxF protein at 67 kDa and two subforms of HoxU at approximately 27 and 23 kDa.

For HoxFU purification, plasmid pGE553 was transferred to *R. eutropha* HF424, which is a mutant derivative that does not synthesize the energy-generating membrane-bound and soluble hydrogenase [24]. The resulting transconjugant cells were grown in liquid fructose-glycerol minimal medium to the late stationary phase. *Strep*-tagged HoxFU heterodimer was purified to homogeneity from the soluble cell extract by means of *Strep*-Tactin affinity and subsequent size exclusion chromatography. The second purification step was efficient in removing inactive HoxFU conformations and a non-related protein of ca. 60 kDa (Figure 2). HoxFU eluted in a prominent peak at an apparent molecular mass of 110 kDa. From 32 g cells (wet weight), we routinely obtained 0.5 mg of HoxFU with high activity (Table S1).

The two protein bands with molecular masses of approximately 23 and 27 kDa (Figure 2) could be unambiguously assigned to HoxU by peptide mass fingerprinting (Table S2). Both subforms still contained the original N- and C-termini, which excludes proteolysis as the reason for the different electrophoretic migration properties and suggests a yet unidentified protein modification.

On the basis of SDS- PAGE analysis, no protein bands attributable to HoxI were observed in the purified HoxFU samples (Figure 2), confirming that the HoxI subunits dissociate from HoxFU during the purification process, which employed a higher ionic strength (150 mM KCl) and slightly alkaline conditions (pH 8.0) [6].

### Biochemical characterization of purified HoxFU

The NADH:benzyl viologen (BV) oxidoreductase activity of the HoxFU preparation was determined at pH values ranging from 6–11 (Figure S4). Maximum activity was reached at pH 10. Notably, the pH optimum for H_2_-dependent NAD^+^ reduction catalyzed by the native SH is pH 8.0 [7]. However the HoxFU-mediated NADH oxidation activity at pH 10 was significantly diminished after approximately 30 s indicating protein instability at this non-physiological pH.

In order to compare the HoxFU activities with that of native SH and Complex I, all subsequent kinetic studies were carried out at pH 8.0. A value of 882 µM was determined for the Michaelis-Menten constant (*K*_M_) for the artificial electron acceptor BV (Figure S5). The HoxFU-mediated turnover rate for the NADH: BV oxidoreductase activity was 639 s^−1^, and the apparent *K*_M_ value for NADH was calculated to be 56 µM (Figure S6). The turnover frequencies and *K*_M_ values of HoxFU are comparable to those of native SH [7] indicating that the diaphorase active site does not suffer upon detachment from the hydrogenase module of the SH.

Pre-incubation of HoxFU for 15 min with NADH at concentrations exceeding the *K*_M_ led to a significant decrease in activity (Table 2). This was consistent with the release of the HoxFU-bound FMN into the supernatant as determined by fluorescence spectroscopy. Minimal background FMN release, even in the absence of NADH, can be explained by mechanical and slight temperature changes in the course of the centrifugation process. An excess of free FMN in the assay prevented the inactivation and even increased the activity of the HoxFU module. A similar effect has previously been observed for native SH [7], [15].

**Table 2:** NADH-mediated reduction leads to the release of FMN and concomitant inactivation of the HoxFU moiety.

		Specific activity [U/mg]^a^	
NADH [µM]	FMN release [%]^b^	− FMN	+ FMN
0	16±2	294±8	356±0
25	29±3	297±41	371±6
100	62±3	138±35	356±22
500	96±4	59±10	246±41

a Purified HoxFU was incubated with various NADH concentrations in the presence (500 µM) and absence of exogenous FMN. After 15 min, benzyl viologen was added and the specific NADH→BV activity was determined.

b The release of FMN from the HoxFU module (without addition of exogenous FMN) was determined by analyzing the HoxFU filtrate from centrifugal filter devices (3 kD cut-off size, 4°C, 7 minutes at 14000× g Millipore, Bedford, MA, USA) by fluorescence spectroscopy.

### Cofactor analysis in HoxFU

Fluorescence determination revealed 0.8–0.9 FMN per HoxFU unit, and the FMN in the catalytically active HoxFU protein showed typical spectrofluorometric emission and excitation spectra (Figures S7,S8) [7]. Using inductively coupled plasma optical emission spectrometry, 11–13 Fe per FMN were detected. This is close to the 14 Fe atoms predicted for HoxFU on the basis of conserved iron sulfur cluster coordination sites that are involved in Fe-S cluster coordination in Complex I (Figures S1,S2,S3).

**Figure 3:**
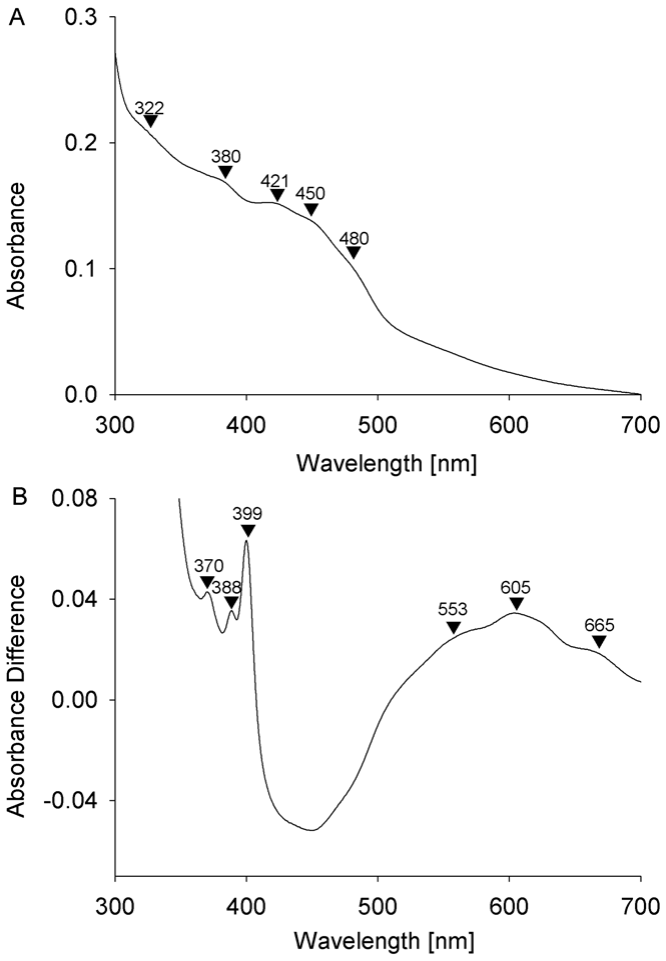
UV/vis spectra of oxidized (as-isolated) and reduced HoxFU. Panel A shows the spectrum of an as-isolated sample (4.8 µM). Prominent peaks and shoulders are indicated by arrows (see text for assignment). Panel B shows the difference spectrum of dithionite reduced (330 µM) minus as-isolated samples.

The content and redox activity of cofactors in HoxFU was further analyzed by UV/visible spectroscopy. Figure 3 (panel A) shows broad shoulders at around 380 nm and 450 nm which can be attributed to FMN in its oxidized form [26], [27]. Additional shoulders at 322/380 nm and 421/480 nm are consistent with the presence of [2Fe-2S] and [4Fe-4S] clusters, respectively [28], [29], [30]. On the basis of these results and the homology to the Nqo1, Nqo2 and Nqo3 subunits of Complex I from *T. thermophilus* (Figures S1,S2,S3), we assign one FMN, one [2Fe-2S] cluster and three [4Fe4S] clusters as the cofactor constituents of the HoxFU modules from *R. eutropha*. The high similarity of the absorbance spectrum of oxidised *R. eutropha* HoxFU to that of the HoxFU subcomplex from *Rhodococcus opacus* [17] suggests that these proteins have the same cofactor composition.

Dithionite-reduced HoxFU samples display shoulders at 553 nm, 605 nm and 665 nm (Figure 3, panel B), which are found for neutral semiquinone radicals [31], [32], [33], [34], [35], [36]. We cannot exclude contributions from [Fe-S] clusters to these signals. However, due to the low extinction coefficients of [2Fe-2S] and [4Fe-4S] clusters in the reduced state, they should be minor. The additional signals at 370 nm 388 nm and 399 nm can be attributed to the anionic semiquinone radical form of the flavin [31], [34], [35], [36]. So far the anionic semiquinone level of the flavin has not been reported for the native SH from *R. opacus* or *R. eutropha* [17] suggesting that this state is only formed at low potentials that can be experienced in isolated HoxFU.

Using an extinction coefficient of 12,500 M^−1^ cm^−1^ for FMN at 450 nm (the value was chosen because it represents an average of the extinction coefficients for bound FMN in various proteins ranging between 10,500–15,400 M^−1^ cm^−1^ [37]), 0.9 FMN per HoxFU protein was calculated from the difference spectrum. This shows that the FMN in HoxFU was reduced quantitatively by dithionite.

### Production of reactive oxygen species

For quantification of superoxide .production by HoxFU in the presence of NADH and O_2_, we used an established assay that exploits the superoxide-dependent oxidation of hydroxylamine to nitrite [21]. In order to avoid HoxFU inactivation, measurements were done in the presence of 25 µM NADH which is below *K*_M_ and prevented inactivation and concomitant FMN release from HoxFU upon reaction with NADH. The HoxFU module generated 53.5 nmol superoxide per mg per min at ambient O_2_ (300 µM [38]) corresponding to a turn-over rate of 5.9 min^−1^ (Table 3). Thus, the HoxFU-mediated O_2_–reduction activity was more than 6500-fold lower than the BV reduction activity (349 µmol min^−1^ mg^−1^). However, in fully O_2_-saturated buffer (∼1.4 mM O_2_), the superoxide production increased by 8–9 fold. The addition of superoxide dismutase essentially abolished the liberation of superoxide confirming the specificity of the assay (Table 3).

**Table 3:** NADH-mediated superoxide production by HoxFU in the presence of O_2_ a.

	Superoxide production	
	Specific activity	Turnover rate
O_2_ concentration in the assay [µM]	[nmol/min/mg]	[min^−1^]
300 µM (air-saturated)	53.5±7.4	5.9±0.8
1428 µM (100% O_2_)	465.0±23.9	51.2±2.6
1428 µM (100% O_2_)+650 Units SOD^b^	13.0±4.9	1.4±0.5

a HoxFU (4.9 pmol) was incubated with 25 µM NADH at two different O_2_ concentrations. Superoxide production was measured spectrophotometrically on the basis of hydroxylamine oxidation.

b One Unit of superoxide dismutase (SOD) is defined as the amount of SOD required to inhibit the rate of xanthine oxidase-mediated reduction of cytochrome *c* by 50% [17]. Values represent the arithmetic means and standard deviations of three independent experiments.

### Electrochemical analysis

**Figure 4:**
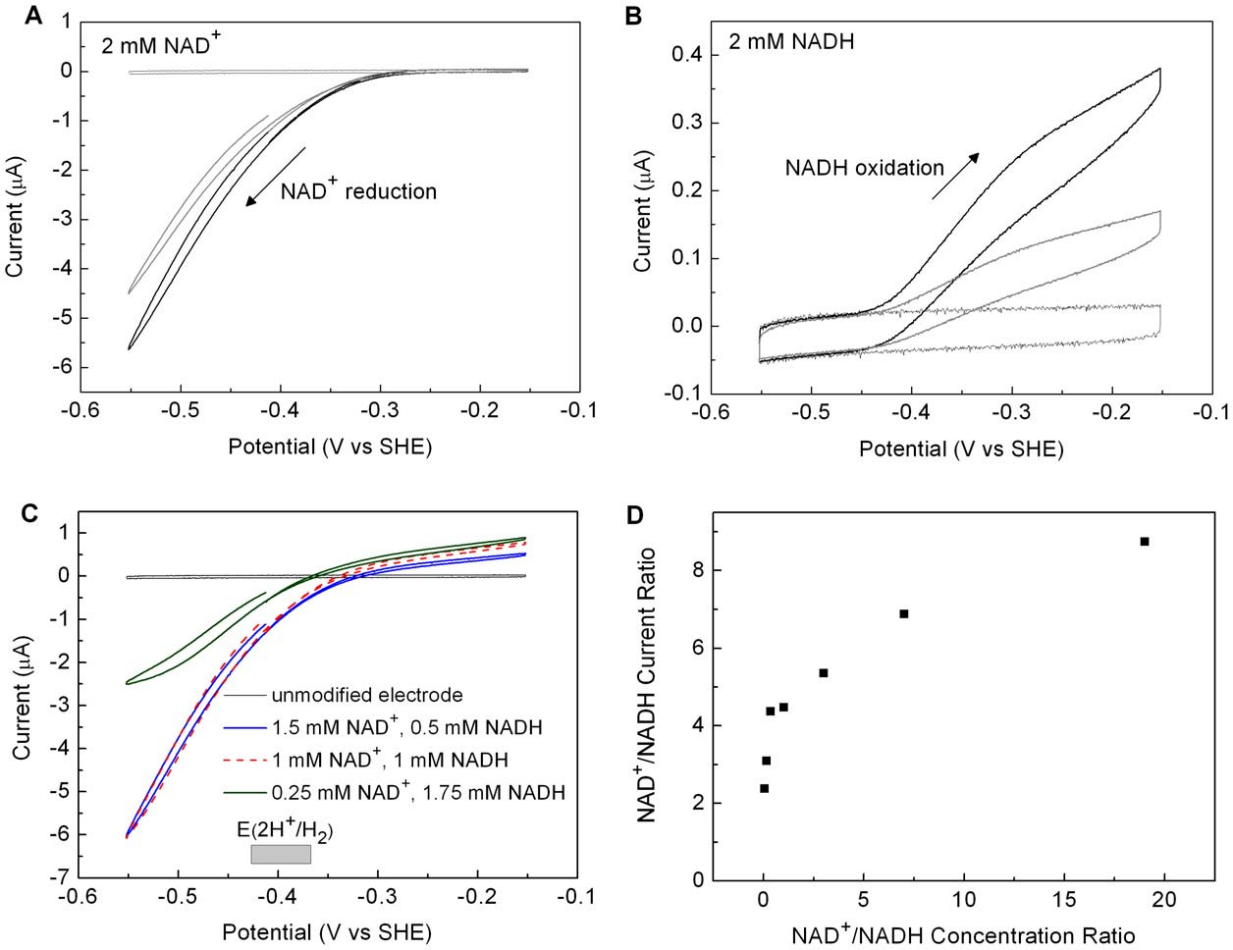
Cyclic voltammograms for an electrode modified with HoxFU recorded at different ratios of NAD^+^/NADH. (A) 2 mM NAD^+^; (B) 2 mM NADH; (C) at concentrations as indicated. In panels A and B, the first (black) and second (gray) cycles recorded after preparing a fresh film on the electrode are shown. In panel C, only the first cycle is shown for each ratio of NAD^+^/NADH. Other conditions: Tris-HCl buffer (50 mM, pH 8.0) 30°C, scan rate 10 mV/s, electrode rotation rate: 2500 rpm. In panel C, the shaded box indicates the range of potentials for *E*(2H^+^/H_2_) at pH 8.0 and 30°C between 100 nM and 10 µM H_2_. Voltammograms in A and C were commenced from −440 mV and in B were commenced from −550 mV, with the potential swept first towards more positive values. Panel D: average current magnitude at 140 mV more negative than *E*(NAD^+^/NADH) (NAD^+^ reduction) over the current at 140 mV more positive than *E*(NAD^+^/NADH) (NADH oxidation) plotted against the ratio of substrate concentrations (2 mM total NAD^+^ and NADH).

Figure 4 shows voltammograms recorded at an electrode modified with HoxFU in Tris-HCl buffer solution (50 mM, pH 8.0) at 30°C which show that the diaphorase moiety catalyses both NAD^+^ reduction at low potentials and NADH oxidation at higher potentials. The electrode is rotated rapidly to provide an efficient supply of substrate and removal of product at the electrode. No Faradaic current is observed for an *unmodified* electrode in solutions containing NAD^+^ and NADH over the experimental potential range (−600 to +250 mV). The voltammograms in Figure 4 were recorded at different ratios of NAD^+^ : NADH with the total concentration [NAD^+^]+[NADH] kept at 2 mM; these concentrations are likely to cover the range found in the cytoplasm under normal cell conditions [39]. At 2 mM NAD^+^ (panel A), a well-defined electrocatalytic wave corresponding to NAD^+^ reduction by HoxFU commences at about −280 mV. At 2 mM NADH (panel B), oxidation of NADH commences at *ca* −450 mV. The drop in catalytic current between the first and second cycles is likely to arise from slow dissociation of the protein film from the electrode, but could also be due to protein denaturation or loss of activity due to dissociation of FMN. Figure 4C shows voltammograms recorded at different mixtures of NAD^+^ and NADH. The catalytic current now crosses the current axis close to the thermodynamic potential for *E*(NAD^+^/NADH) for each set of conditions (shifting by 30 mV with each decade of change in [NAD^+^]/[NADH]). Potentials for the 2H^+^/H_2_ couple in the range 100 nM–10 µM H_2_ (pH 8.0, 30°C) are marked on panel C (shaded box). Thermodynamically efficient catalysis of NAD^+^ reduction and NADH oxidation by HoxFU maximizes the driving force for coupling these reactions to H_2_ oxidation and H^+^ reduction respectively. Each trace in Figure 4C was performed on a fresh film of HoxFU. Variation in protein coverage from one film to another means that the magnitude of the current is not a reliable indicator of the absolute activity at each substrate concentration. However, the relative current (activity) for NAD^+^ reduction vs NADH oxidation in each voltammogram, measured at 140 mV on either side of the zero current potential (panel D), indicates the catalytic bias at each set of conditions. At all NAD^+^/NADH ratios sampled in this set of experiments the catalytic activity is higher for NAD^+^ reduction than NADH oxidation.

In the absence of substrate, cyclic voltammograms for HoxFU show peaks at ca 280 mV on the forward and return scans characteristic of a surface adsorbed species (Figure S9). Free FMN adsorbs strongly onto a graphite electrode, giving rise to peaks at the same potential. It is thus likely that the features observed for HoxFU arise from flavin cofactor that has dissociated from the protein and adsorbed onto the graphite, rather than electron transfer to and from flavin within the protein. The peaks were not observed in catalytic voltammograms recorded in the presence of NAD^+^ and/or NADH (Figure 4).

### Electrochemical determination of *K*_M_ for NADH oxidation and NAD^+^ reduction

**Figure 5:**
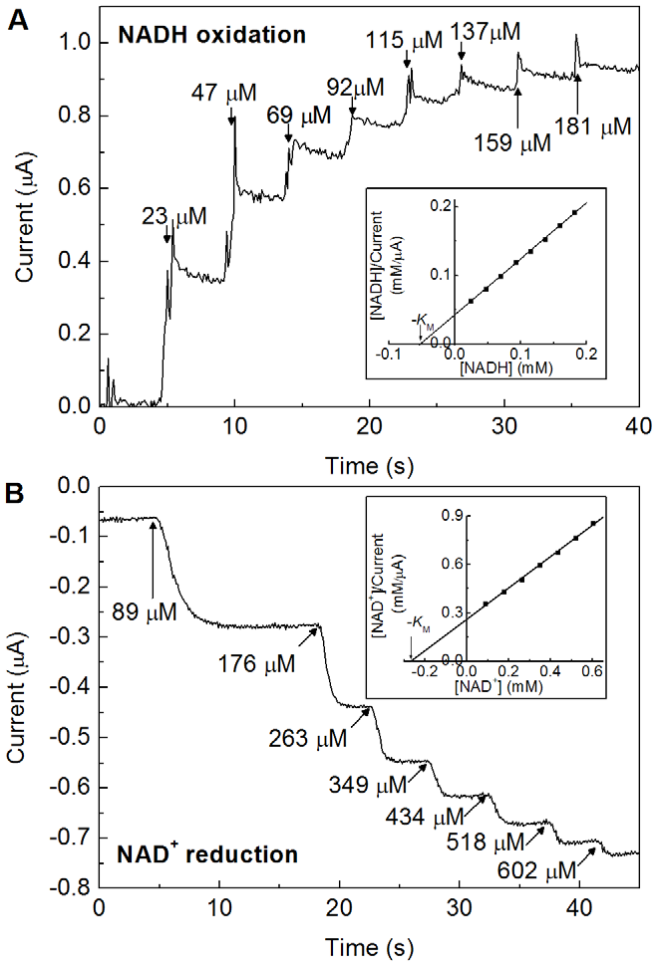
Electrochemical experiments designed to measure values of *K*_M_ for NADH oxidation and NAD^+^ reduction. (A) at −62 mV, NADH concentrations as indicated. (B) at −412 mV, NAD^+^ concentrations as indicated. Other conditions: Tris-HCl buffer (50 mM, pH 8.0), 30°C, electrode rotation rate: 2500 rpm. The inset panels show Hanes / Woolf plots for determination of *K*_M_; current values in (B) were corrected for a non-Faradaic current offset which is evident at zero substrate.

We next used electrochemical experiments to determine Michaelis-Menten constants for NADH oxidation, *K*_M(NADH)_, and NAD^+^ reduction, *K*_M(NAD_+_)_. Figure 5A shows an experiment in which the electrode is held at a constant potential of −62 mV while the concentration of NADH is increased by injections into the solution. The inset shows a plot of [NADH]/current vs [NADH]. Since the current is directly proportional to catalytic activity, this plot is analogous to a Hanes or Woolf plot [40], [substrate]/activity vs [substrate], such that the x-intercept = −*K*_M_. This experiment was performed 6 times, giving a mean value for *K*_M(NADH)_ of 58±18 µM, comparable to the value obtained from solution experiments (56 µM) with BV as electron acceptor. Figure 5B shows an analogous experiment for determination of a value for *K*_M(NAD_+_)_, in which the electrode is held at a constant potential of −412 mV while the concentration of NAD^+^ is increased. This experiment was repeated 9 times, yielding *K*_M(NAD_+_)_ = 197±28 µM. Following the first addition of NAD^+^ (89 µM) to the substrate-free solution, the current magnitude increases slowly (Figure 5B) whereas the response on addition of NADH is fast (panel A). The slow response to NAD^+^ is therefore not merely due to solute mixing and suggests that a structural rearrangement occurs when enzyme molecules first encounter NAD^+^.

### Product inhibition of NADH oxidation and NAD^+^ reduction

**Figure 6:**
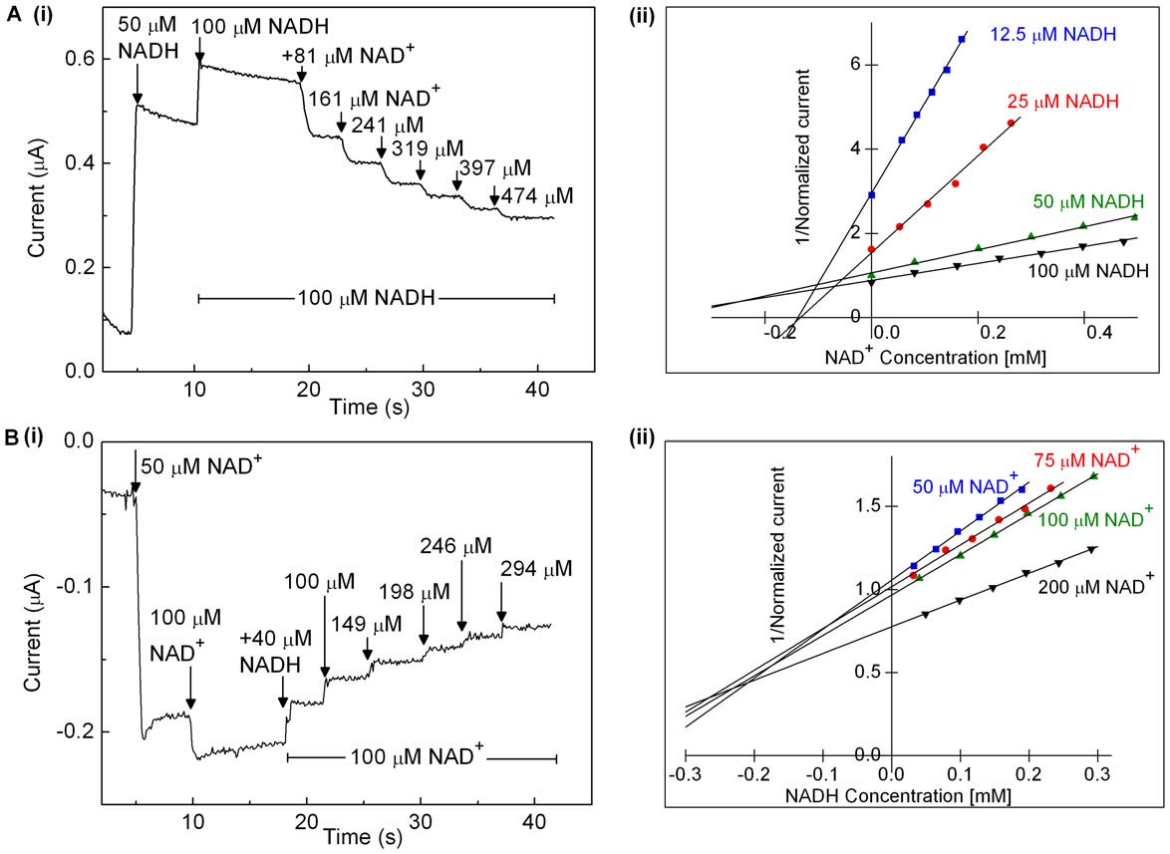
Demonstration of product inhibition of HoxFU. Raw data for experiments examining NADH oxidation at −62 mV (A) and NAD^+^ reduction at −412 mV (B) are shown in plot (i) in each case. The substrate concentration was zero at the start of each experiment, and 50 µM substrate was then injected as indicated to provide a control step for normalization of the catalytic activity of each film. The substrate concentration was then adjusted to x µM (by dilution or addition as required. In the experiments shown in panels labelled (i), x = 100. Aliquots of the inhibitor were then injected from a stock solution containing x µM substrate in order to keep the substrate level at x µM throughout the remainder of the experiment. Panels labelled (ii) show Dixon plots presenting data from a series of such experiments. Experiments were performed at 30°C, electrode rotation rate: 2500 rpm.

The results in Figure 6A show that NADH oxidation by HoxFU is inhibited by product, NAD^+^. In the experiment shown in panel A(i), NADH is first introduced at a standard concentration, 50 µM, to allow us to compare currents from experiments performed on different films. A further injection of NADH was then made to take the concentration to 100 µM. Injections from a stock solution of NAD^+^ (also containing 100 µM NADH) were then performed as indicated, each addition leading to a drop in current. Increasing the proportion of NAD^+^ while holding the electrode at a fixed potential relative to SHE is expected to lead to a small drop in catalytic current due to the slightly diminished driving force for NADH oxidation relative to *E*(NAD^+^/NADH). However the drop in current that follows each NAD^+^ addition is too large to be attributed just to the change in driving force. To determine a value for the inhibition constant, *K*_I(NAD_+_)_, the experiment was repeated at different final NADH concentrations, either by diluting the initial solution or adding more NADH. In each case, the current was corrected for a linear offset arising from the electrode, and the catalytic current for each film was normalized by dividing by the current at the 50 µM NADH. Analysis of the data is also complicated by background loss of enzyme activity, due to slow dissociation of protein from the electrode surface, or damage to the enzyme, and therefore cannot be modelled by a simple function. The set of experiments was repeated several times at different substrate concentrations, and plot (ii) in Panel A shows a typical set of results in the form of a Dixon plot. For competitive inhibition, the lines should intersect at the point where −[substrate] is equal to *K*_I_. The lines intersect in the region of −0.1 to −0.3 mM, suggesting that *K*_I(NAD_+_)_ is in the range 0.1 to 0.3 mM. Figure 6B shows similar experiments for inhibition of NAD^+^ reduction by NADH. The lines in the Dixon plot (ii) intersect more clearly at around −0.2 to −0.3 mM NADH, indicating a *K*_I(NADH)_ value of ca 0.2–0.3 mM - a similar range to *K*_I(NAD_+_)_. Dixon plots from data in which exponential decay functions have been applied in an attempt to correct for background loss of activity yield very similar values for *K*_I_.

### Inactivation at low potentials: substrate concentration effects

**Figure 7:**
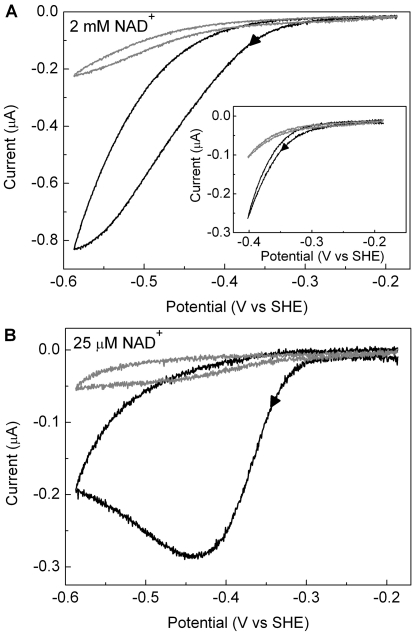
Cyclic voltammograms showing irreversible inactivation of HoxFU at low potentials at different NAD^+^ concentrations. In each case the scan rate was 1 mV/s, the electrode was rotated at 2500 rpm, and other conditions were: 50 mM Tris-HCl buffer, pH 8.0, 30°C; (A) 2 mM NAD^+^ and (B) 25 µM NAD^+^. The first cycle for each film is shown as a thick line and the second cycle is shown as a thin line. Arrows indicate the direction of scan. Inset in (A) shows a scan over a narrower potential range.

The cyclic voltammograms in Figure 7 were recorded at a slow scan rate during reduction of NAD^+^ by HoxFU and show irreversible loss of activity at low potentials. During these slow scans, there is considerable background loss of enzyme activity attributed to dissociation of protein from the electrode. However, an additional effect is observed in cycles with a lower potential limit which is more negative than about −0.4 mV: considerable hysteresis is observed in the catalytic current for the forward and reverse potential sweeps (see Figure 7B) and the second cycle shows a shift in the onset for catalysis towards more negative potentials. This is confirmed by examination of the derivative plot, d*i*/d*E* vs *E* (not shown). This effect becomes more pronounced as the concentration of NAD^+^ is decreased, and at 25 µM NAD^+^ activity is lost almost completely during the forward sweep. The experiment was repeated keeping a constant total concentration of [NAD^+^]+[NADH] (2 mM), but the extent of irreversible inactivation was indistinguishable (data not shown).

### Activity in the presence of O_2_

**Figure 8:**
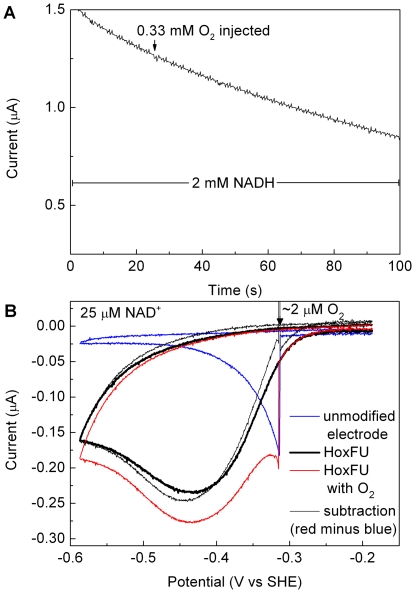
Ability of HoxFU to (A) oxidize NADH and (B) reduce NAD^+^ in the presence of O_2_. In panel A, the potential was held at 243 mV so that the enzyme film oxidises NADH. The drop in current throughout the experiment is attributed to enzyme dissociation from the electrode. An aliquot of air-saturated solution was introduced as indicated. Panel B shows a series of cyclic voltammetric experiments recorded at a scan rate of 1 mV/s with the electrode rotated at 2500 rpm in a solution of 50 mM TrisHCl pH 8.0 containing 25 µM NAD^+^. The solid black line shows the response for a HoxFU film as the potential is swept from −0.2 V to −0.6 V. The blue line shows the response for an unmodified electrode, as O_2_ is introduced into the solution during the forward scan as indicated. The red line shows the response when the same amount of O_2_ is introduced during a potential cycle for an electrode modified with a fresh film of HoxFU (normalized to the current magnitude of the black forward trace at −300 mV). Subtraction of the blue trace (contribution from direct O_2_ reduction) from the red trace yields the thin black trace which is similar in shape to the solid black trace, confirming that the characteristic shape of the HoxFU electrocatalytic wave is retained in the presence of O_2_.

Figure 8 shows experiments which report on the activity of HoxFU in the presence of O_2_. The experiment shown in Panel A was carried out in a solution of 2 mM NADH, and the potential was held at +243 mV in order to drive NADH oxidation by the enzyme while avoiding direct reduction of O_2_ at the graphite electrode. Some background loss in current is observed due to instability of the enzyme film. At 25 s, an aliquot of buffer containing 2 mM NADH (to avoid substrate dilution) and saturated with O_2_ was injected into the cell solution to give an O_2_ concentration of *ca* 0.33 mM. No drop in current in response to O_2_ was detected, indicating that oxidation of NADH by HoxFU is not significantly inhibited by O_2_.

The cyclic voltammograms in panel B (all recorded at 25 µM NAD^+^) demonstrate the ability of HoxFU to reduce NAD^+^ in the presence of O_2_. Experiments in this potential regime are more complicated because of direct reduction of O_2_ at the electrode, but we use the characteristic shape of the HoxFU electrocatalytic wave (solid black trace) to confirm HoxFU activity. In the red and blue traces, an aliquot of O_2_-saturated buffer (containing NAD^+^) was injected at approximately −310 mV on the sweep towards negative potentials; the O_2_ is then flushed out of solution by the rotating electrode during the course of the scan. For an unmodified electrode (no HoxFU, blue trace), the O_2_ injection leads to a rapid increase in reductive current followed by a decay in current as the O_2_ concentration diminishes. For a HoxFU electrode (freshly prepared film, red), introduction of O_2_ gives rise to an increase in current due to O_2_ reduction superimposed on enzyme-catalyzed NAD^+^ reduction; the total current continues to rise as the driving force for NAD^+^ reduction increases, but slowly returns to the level expected for the enzyme under fully anaerobic conditions as O_2_ is flushed out. Subtraction of the blue trace (contribution from direct O_2_ reduction) from the red HoxFU trace yields the thin black line which is similar in shape to the solid black trace, confirming that the characteristic shape of the HoxFU electrocatalytic wave is retained in the presence of O_2_. (Variations in capacitive current and O_2_ reduction current for bare graphite *versus* the enzyme-modified electrodes cause slight errors in the subtraction, so the traces do not overlay perfectly.) The ability of HoxFU to reduce NAD^+^ in the presence of O_2_ is further confirmed by an experiment in which HoxFU is inhibited by ADP-ribose in the presence and absence of O_2_ (Figure S10).

## Discussion

In this work, we report the homologous overproduction and isolation of the HoxFU diaphorase subcomplex of *R. eutropha* O_2_-tolerant NAD^+^-reducing soluble hydrogenase. The subcomplex is catalytically active in transferring electrons from NADH to benzyl viologen, and exhibits electrocatalytic NADH oxidation and NAD^+^ reduction on a graphite electrode. This has allowed us to characterize the diaphorase activity of the SH in isolation from its hydrogen cycling moiety and full electron transfer chain. We have therefore been able to examine the catalytic bias of the NAD^+^/NADH site, its ability to function in the presence of O_2_, and its production of ROS under aerobic conditions. These results are interpreted in the context of the behavior of the intact hexameric SH, the physiological role of the enzyme, and its relationship to Complex I.

### Direction of catalysis and possible control mechanisms

No crystallographic structure is available for an NAD^+^-reducing hydrogenase, but a high-resolution X-ray structure of the soluble domains of *T. thermophilus* Complex I shows NADH stacked next to the FMN of Nqo1 (analogous to FMN-b of the SH) in an orientation compatible with direct hydride transfer from NADH to FMN [41]. It is highly likely that oxidation of NADH in the SH also proceeds via direct hydride transfer to oxidized FMN. Electrons must then be transferred one at a time from the reduced FMN to the FeS clusters or artificial electron acceptors (here, BV). At least two alternative reaction pathways are possible: (1) The FMN site is re-oxidized by an electron acceptor before dissociation of NAD^+^ or (2) NAD^+^ may dissociate from the reduced binding site, leaving an electron acceptor to re-oxidize the product-free enzyme. A simple hyperbolic initial rate is observed for NADH oxidation by BV (Figure S6), which, according to steady-state kinetics, is consistent with the behavior expected for route (1). The reverse reaction, reduction of NAD^+^, observed for HoxFU on an electrode, is likely to proceed via hydride transfer from reduced flavin to NAD^+^, although the series of steps during catalysis need not necessarily represent the reverse of steps in NADH oxidation since the reactions are driven at different potentials (energies).

When *R. eutropha* grows under aerobic growth conditions in the presence of H_2_, the normal direction of catalysis for the SH is NAD^+^ reduction by H_2_. The majority of NADH produced by the SH will be used by Complex I to establish a proton motive force, while some NADH will be converted by transhydrogenases to NADPH which is required for anabolic metabolism [42]. It has been reported that the SH may also act as an electron valve, working in the reverse direction (NADH oxidation coupled to H^+^ reduction) under conditions where the NADH/NAD^+^ pool has become too reduced [9]. In particular, short-lived NADH oxidation coupled to H^+^ reduction may be important in balancing the NAD^+^/NADH pools when *R. eutropha* is shifted from aerobic to anaerobic growth conditions [4], [9]. Similarly, in Complex I, where the normal function is in NADH oxidation, reverse electron flow can occur under certain conditions to reduce NAD^+^ [43]. Additionally, the ability of the SH to channel electrons from NADH to the NiFe hydrogenase active site could be important in re-activating oxidized inactive states of the Ni-Fe centre [7], [16]. The bidirectionality of the HoxFU module agrees with previous observations on the cognate HoxHY hydrogenase moiety of the SH [16]. However, while native SH and HoxFU are biased towards H_2_ oxidation and NAD^+^ reduction, respectively, the isolated HoxHY heterodimer showed a slight preference for proton reduction. The detachment of HoxHY from the SH holoenzyme resulted in a significant decrease of both the H_2_ oxidation and H^+^ reduction activities preventing a firm conclusion on the catalytic bias of this module. By contrast, the HoxFU moiety showed catalytic activities comparable to those of the native SH, minimizing the risk of misinterpretation by studying a genetically constructed subcomplex.

The affinity constants of HoxFU for NADH and NAD^+^, and product inhibition of both NADH oxidation and NAD^+^ reduction may serve as control mechanisms to balance these reactions. The value of *K*_M(NADH)_ determined for HoxFU in this study (56 µM) is of the same order of magnitude as that of bovine Complex I (90 µM) [44]. This suggests that the SH is capable of functioning in the NADH oxidation direction under physiological conditions. However, assuming that the NAD^+^/NADH ratio under aerobic conditions in *R. eutropha* is approximately 10∶1, as previously determined for *E. coli* [39], and taking into account the product inhibition at physiologically relevant levels of NAD^+^ (*K*_I(NAD_+_)_, ca 0.1–0.3 mM), catalytic NADH oxidation by the SH under oxic conditions is rather unlikely. This may represent a control mechanism to avoid excessive loss of reducing power from the cell in the form of H_2_ which escapes readily.

Electrocatalytic NAD^+^ reduction is clearly observed in PFE studies. These yield a value of 197±28 µM for *K*_M(NAD_+_)_, about 2.5× lower than the value obtained for H_2_:NAD^+^ activity in the intact SH (500 µM) [7]. Interestingly, these values are significantly higher than *K*_M(NAD_+_)_ for bovine mitochondrial Complex I (7 µM) [45]. The relatively low affinity of the SH for NAD^+^ and significant product inhibition of NAD^+^ reduction (*K*_I(NADH)_ ca 0.2–0.3 mM) would slow the rate of H_2_:NAD^+^ activity, perhaps assisting NADH oxidation under conditions where the NADH/NAD^+^ pool is too reduced (i.e. by hindering the back reaction).

Protein film electrochemistry experiments on HoxFU confirm that NAD^+^ reduction and NADH oxidation occur very close to *E*(NAD^+^/NADH) (corrected for the experimental conditions), i.e. HoxFU operates in either direction with minimal overpotential. This would be critical to the function of the SH because *E*(H^+^/H_2_) is very closely spaced in potential to *E*(NAD^+^/NADH) leaving minimal driving force for either H_2_:NAD^+^ or NADH:H^+^ activity. Voltammetric experiments conducted over a range of NAD^+^/NADH ratios (with total concentration 2 mM) reveal that catalysis by HoxFU is most active in the direction of NAD^+^ reduction over this range, despite *K*_M(NAD_+_)_ being higher than *K*_M(NADH)_. Although we are unable to determine the catalytic constant, *k*_cat_, from PFE experiments because we do not know the electroactive coverage for each enzyme film, *k*_cat_ must be higher for NAD^+^ reduction relative to NADH oxidation in order for the observed current magnitude (activity) to be higher for NAD^+^ reduction for a given film (Figure 4C). Thus the SH would be effective in NAD^+^ reduction by H_2_ under normal operating conditions in the cell despite the relatively high *K*_M(NAD_+_)_. Furthermore, continual consumption of NADH produced by the SH, either by Complex I or transhydrogenases, will keep the NAD^+^/NADH pool relatively oxidized. The diaphorase moiety of the SH thus appears to be finely tuned to balance the NAD^+^/NADH ratio within tight limits.

### Potential-dependent effects on activity and FMN release

PFE studies by Hirst and coworkers showed that NAD^+^ reduction by a sub-complex of Complex I from bovine mitochondria comprising the 51 and 24 kDa subunits (analogous to the subunits Nqo1 and Nqo2 of Complex I from *T. thermophilus*) undergoes a reversible reductive inactivation process at potentials close to that of the FMN centre (ca −0.4 V) [45]. Two models gave simulated voltammograms consistent with the experimental data, one involving enhanced activity at the semi-reduced level of the flavin, and the other model invoking effects of redox state of the nearby 2Fe-2S cluster in the 24 kDa subunit (analogous to Nqo2). It was suggested that this potential-dependent switch could have a physiological role in hindering reverse electron transport (ubiquinol:NAD^+^) in Complex I [46]. We did not observe a sharp switch-off/on in electrocatalytic NAD^+^ reduction by HoxFU of the SH at NAD^+^ concentrations above *K*_M(NAD_+_)_. The equivalent of the 24 kDa subunit 2Fe-2S cluster is absent in HoxFU, lending support to the involvement of this cluster in Complex I activity switch during NAD^+^ reduction. Slow loss of HoxFU activity *is* observed at negative potentials, particularly at NAD^+^ concentrations well below *K*_M(NAD_+_)_ (see the voltammogram in Figure 7B recorded at 25 µM NAD^+^), but there is no evidence for oxidative recovery of activity when the potential is swept back to positive values, and this effect is likely to be due to reductive damage to the protein. Reducing conditions were previously described to lead to the loss of FMN-a in the SH or the corresponding flavin in Complex I, particularly in the absence of NAD^+^ [15], [47]. The hysteresis observed on sustained treatment at potentials below about −0.4 mV and the shift in the onset for catalytic NAD^+^ reduction (Figure 7) suggests that the remaining activity is associated with a damaged fraction of enzyme. Simulation of the voltammetric response for Complex I also required consideration of enhanced denaturation or FMN loss at negative potentials. We were unable to determine the potential for FMN in HoxFU, but by analogy with the potential of the flavin in Complex I, its 2e^−^ reduction potential is likely to be around −0.4 V, consistent with the potential at which reductive loss of activity is observed. At concentrations of NAD^+^ well below *K*_M(NAD_+_)_, the flavin site spends significant time unoccupied; when the electrode is driven to low potentials in PFE studies, the FMN will be readily reduced and lost. In solution studies, high NADH concentrations were also found to cause loss of activity related to FMN release, and this can be explained by the reducing conditions imposed by a high NADH:NAD^+^ ratio. The stability of the catalytic current for NAD^+^ reduction and NADH oxidation by immobilised HoxFU seems to depend on the relative concentrations of the two substrates (see for example Figure 6), suggesting that bound substrate stabilises the enzyme to some extent. Further work to determine the midpoint potentials for redox centres in HoxFU using EPR redox titrations will be informative in making further comparisons to Complex I.

### Role of FMN for superoxide production

In Complex I, reduced flavin [48] and a second site, the 4Fe4S cluster known as N2 in Nqo6 or a semiquinone [21], [49] are recognized as important contributors to superoxide (O_2_^•−^) production, especially during reverse electron transfer (ubiquinol:NAD^+^) or at high NADH/NAD^+^ ratios. In the presence of NADH, HoxFU produced superoxide with a turnover rate of 5.9 min^−1^, which is higher than the corresponding rates of the SH holoenzyme (0.5 min^−1^), glutathione reductase (0.8 min^−1^), but moderate to low compared to succinate dehydrogenase (13 min^−1^), bovine heart Complex I (40 min^−1^), and fumarate reductase (1,600 min^−1^) [21], [48], [50]. However, we assign HoxFU as the major source of ROS in the entire SH. Direct hydride transfer between NADH and FMN will lead to the 2e^−^-reduced state of FMN, but single electron transfers to the Fe-S clusters will generate semiquinone radical states of the cofactor, and we observed semiquinone radical formation upon reduction of HoxFU by dithionite or NADH. In Complex I, it has been suggested that the 2Fe-2S cluster in the 24 kDa subunit may minimize the lifetime of the reduced flavin by transiently accepting an electron [22], and the absence of an analogue to this cluster in HoxFU may be a contributor to the high production of O_2_^•−^. In the intact SH, the electron transport chain and the ready availability of H^+^ for reduction at the NiFe hydrogenase active site is also likely to support rapid electron transfer away from reduced flavin. (In PFE experiments in which O_2_ is introduced during electrocatalytic NADH oxidation, the electrode provides a sink for rapid removal of electrons from reduced FMN via the FeS clusters, and we would not expect to observe significant O_2_^•−^ formation.)

### Ability of the SH to function under aerobic conditions

The PFE experiments on HoxFU show that its activity in either direction (NAD^+^ reduction or NADH oxidation) is retained in the presence of O_2_. This is consistent with the requirement for the SH to function under aerobic conditions, although the relatively high rate of superoxide production by HoxFU suggests that some electrons are ‘short-circuited’ at the FMN site and used for partial reduction of O_2_. Since the superoxide production rate of the SH holoenzyme is one order of magnitude lower than that of HoxFU, the short-circuiting might be a result from the detachment of the hydrogenase module.

There is much interest in using hydrogenases (*in vitro* or *in vivo*) for H_2_ production coupled to oxygenic photosynthesis. Thus understanding the mechanism and scope of O_2_ tolerance in hydrogenases able to carry out hydrogen cycling in air is crucial for these applications. Recent spectroscopic studies of the SH in whole cells and the isolated HoxHY moiety indicate a standard set of inorganic ligands, *i.e.* one CO and two CN^−^ [16], [51]. This is in contrast to previous reports that correlated the O_2_ tolerance of the SH to the presence of additional cyanide ligands in the Ni-Fe active site [8], [52], [53], [54]. In PFE experiments on the isolated HoxHY module of the *R. eutropha* SH, we found that O_2_-inactivated states require low-potential electrons for re-activation [16]. In the intact SH, it is likely that electrons for reactivation of the NiFe site can be supplied from NADH oxidation at FMN-b. The fact that HoxFU remains catalytically competent in both directions in the presence of O_2_, means the SH is functional in storing reducing equivalents from H_2_ in the form of NADH under aerobic conditions. The NiFe site is likely to concomitantly suffer attack from O_2_, and some low-potential electrons from NADH will presumably be diverted (in reverse electron flow) to reductively re-activate the NiFe site [16]. Further waste of reducing equivalents is likely to occur at the FMN-b site, where O_2_ can be reduced to O_2_^•−^. Figure 1 (panel B) summarizes our current understanding of superoxide production, regulation, catalytic bias and O_2_-tolerance of the soluble NAD^+^-reducing hydrogenase from *R. eutropha*.

Efficient catalysis of NAD^+^/NADH cycling, at minimal overpotential relative to the thermodynamic potential *E*(NAD^+^/NADH), means that the redox state of the NiFe cofactor is closely coupled to the potential enforced by the NADH/NAD^+^ pool within the cytoplasm. Electrocatalytic cycling of NAD^+^/NADH by adsorbed HoxFU with relatively high turnover frequencies and over a wide potential range, means that the diaphorase moiety of the SH may be a promising system for electrochemical regeneration of NAD^+^/NADH for biotechnological applications.

## Materials and Methods

### Purification and biochemical characterization of HoxFU

For isolation of HoxFU, cells of *R. eutropha* HF424(pGE553) harboring *Strep*-tagged HoxF in a HoxHY^−^ background [6] were cultivated in mineral salts medium containing 0.4% (w/v) fructose or a mixture of 0.2% (w/v) fructose and 0.2% (v/v) glycerol (FGN medium) [55] supplemented with 1 µM NiCl_2_ and 1 µM ZnCl_2_. Large scale cultivation was performed in a 10 L fermenter (Braun Biotech), cells were harvested at an optical density (at 436 nm) of 9 to 11. The cells were washed with 50 mM potassium phosphate (K-PO_4_) buffer, pH 7.0 containing 50 mM succinate and subsequently resuspended in twice their volume of resuspension buffer (50 mM Tris-HCl, 150 mM KCl, 5% glycerol, pH 8.0 containing additional Protease Inhibitor (EDTA-free, Roche)). After two passages through a chilled French pressure cell at 6.2 MPa, the suspension was centrifuged at 100,000× *g* for 45 min. The soluble extract was applied to a 2 mL *Strep*-Tactin Superflow column (IBA), washed with 6 mL of resuspension buffer and eluted with the same buffer containing 5 mM desthiobiotin. The eluate was then concentrated in an Amicon Diaflo cell (YM 30 membrane; Amicon, Witten, Germany) and subjected to gel filtration (Superdex 200, Amersham Biosciences, 120 mL bed volume) The column was run with resuspension buffer at a flow rate of 1 mL/min. The molecular mass of HoxFU was determined in reference to the migration properties of standard proteins with known sizes. The column steps used in this procedure ensured that HoxI dissociated from HoxFU.

The NADH oxidation activity was measured anaerobically at 30°C in 50 mM Tris-HCl buffer (pH 8.0) containing 1 mM NADH, 5 mM benzyl viologen (oxidized), 90 µM dithionite, and 10 to 50 pmol of enzyme. The absorption was monitored spectrophotometrically at 578 nm (Varian Cary 50, ε = 8.9 mM^−1^ cm^−1^ for benzyl viologen).

Protein concentrations were determined according to the method of Bradford [56]. UV/visible spectra were recorded with a Varian Cary 300 at 16°C. SDS-PAGE was performed according to Laemmli [57], [58], and peptide mass fingerprinting was performed as described in reference [59]. The iron content of the diaphorase subcomplex was quantified by ICP-OES analysis (Optima 2100 DV, PerkinElmer Life Sciences) using multiple element standard solution XVI (Merck) as reference. Flavin mononucleotide concentrations were analyzed fluorometrically using the method described by Schneider [7] modified for a 96-well plate reader (Spectramax M4 spectrofluorometer). FMN (73–79% fluorometric grade, Sigma) was used to prepare standards; and these were normalized using a higher purity FMN sample (approximately 95% by HPLC, Sigma). Superoxide formation was quantified by the oxidation of hydroxylamine to nitrite which was subsequently quantified according to a modified protocol of Schneider and coworkers [21]. The reaction was conducted at 30°C in 50 mM K-PO_4_ buffer, pH 7.0, containing HoxFU (0.54 µg) and hydroxylamine hydrochloride (0.5 mM). The formation of nitrite was determined by measuring the absorption at 530 nm at 20 min after the addition of α-naphthylamine (2.33 mM) and sulphanilic acid (6.33 mM). Sodium nitrite was used as standard. When required, superoxide dismutase (SOD, Cu-Zn-SOD from bovine erythrocytes, Sigma) was added.

For complementation experiments *hoxFU* was deleted in *R. eutropha* HF798 [50] using the conditionally lethal plasmid pCH1462 [16], resulting in HF903. For H_2_-dependent NAD^+^-reduction measurements, cells were permeabilized by treatment with 33 mg mL^−1^ cetyl-trimethyl-ammonium bromide (CTAB) as described previously [60].

### Electrochemistry

Experiments were conducted in an N_2_-filled anaerobic glove box (M. Braun or Glove Box Technology ≤1 ppm O_2_). A pyrolytic graphite ‘edge’ (PGE) rotating disk electrode (area 0.03 cm^2^) was used with an electrode rotator (EG&G 636). The counter electrode was a Pt wire, and a saturated calomel electrode (SCE) was employed as the reference. The electrochemical cell was washed with *aqua regia* between sets of experiments to remove traces of FMN and then rinsed thoroughly with water. Potentials were controlled using an Autolab128N or 302N potentiostat (EcoChemie, Netherlands) and were converted to V vs standard hydrogen electrode (SHE) using the conversion *E*(SHE) = *E*(SCE)+242 mV at 25°C (+238 mV at 30°C). To prepare a protein film, the PGE working electrode was first freshly polished with a slurry of α-alumina (1 µm, Buehler), then rinsed and sonicated for 10 s before application of 0.5 µL of protein (0.4 mg mL^−1^) which was allowed to adsorb over 30 seconds. Excess protein solution was then withdrawn from the electrode by pipette. In most cases the electrode was then rinsed in enzyme-free buffer before inserting into the electrochemical cell. No differences in electrochemical response were observed if this washing step was omitted. The temperature in the jacketed cell was maintained at 30°C by water flow from a circulator (Grant). All experiments were carried out in 50 mM Tris-HCl buffer, pH 8.0, prepared using MilliQ water (resistivity >18.2 MΩ cm). When necessary, NADH (Melford) and NAD^+^ (Sigma or Melford) were purified by ion-exchange chromatography on a Q HyperD F column (Pall Corporation), and concentrations of eluant fractions were determined by comparison of A_340_ and A_260_ with those of standard solutions. For *K*_M_ and *K*_I_ measurements, aliquots of NAD^+^ and NADH were injected from stock solutions into the cell solution using small-volume syringes (Hamilton). Errors on electrochemically determined *K*_M_ measurements are indicated as ±1 standard deviation.

## Supporting Information

Figure S1 **Sequence alignment of the C- terminal part of HoxF from the** ***R. eutropha*** **SH with subunits from related hydrogenases, the FdsB subunit of the NAD^+^-dependent formate dehydrogenase of** ***R. eutropha*** **and the Complex I subunits NuoF from** ***R. eutropha*** **and Nqo1 from** ***T. thermophilus*** **.** Cysteine residues involved in coordination of the [4Fe4S] cluster in Nqo1 of *T. thermophilus* [14] which are conserved in all the other proteins are boxed. Abbreviations: *R.e.*, *Ralstonia eutropha*; *R.o.*, *Rhodococcus opacus*; *T.r.*, *Thiocapsa roseopersicina*; *Syncy.*, *Synechocystis* PCC 6803; *Synco.*, *Synechococcus* PCC 7002; *T.th.*, *Thermus thermophilus*. Amino acids residues conserved in all proteins are marked with a *.
(TIF)

Figure S2 **Sequence alignment of the N- terminal part of HoxF from the** ***R. eutropha*** **SH with subunits from related hydrogenases and the Complex I subunits NuoE from** ***R. eutropha*** **and Nqo2 from** ***T. thermophilus*** **.** Cysteine residues involved in coordination of the [2Fe2S] cluster in Nqo2 [14] are indicated by black boxes. The absence of most of these cysteine residues suggests that HoxF of *R. eutropha* does not contain a [2Fe2S] cluster. Abbreviations: *R.e.*, *Ralstonia eutropha*; *R.o.*, *Rhodococcus opacus*; *T.r.*, *Thiocapsa roseopersicina*; *Syncy.*, *Synechocystis* PCC 6803; *Synco.*, *Synechococcus* PCC 7002; *T.th.*, *Thermus thermophilus*. Amino acids residues conserved in all proteins are marked with a *. The N-terminal part of HoxF shares similarities with HoxE from cyanobacterial bidirectional hydrogenases and Nqo2 from complex 1 whereas the C- terminal part of HoxF is homologous to cyanobacterial HoxF proteins and Nqo1 from complex 1. This bipartite nature indicates that of the *R. eutropha* HoxF represents a fusion protein of two Complex I subunits.
(TIF)

Figure S3 **Sequence alignment of the HoxU from the** ***R. eutropha*** **SH with subunits from related hydrogenases, the FdsA subunit of the NAD^+^-dependent formate dehydrogenase of** ***R. eutropha*** **and the N-terminal parts of the Complex I subunits NuoG from** ***R. eutropha*** **and Nqo3 from** ***T. thermophilus*** **.** Cysteine and histidine residues involved in coordination of the [2Fe2S] cluster (black) and the two [4Fe4S] clusters (red+blue) in Nqo3 [14] are highlighted. Amino acids residues conserved in all proteins are marked with a *. Abbreviations: *R.e.*, *Ralstonia eutropha*; *R.o.*, *Rhodococcus opacus*; *T.r.*, *Thiocapsa roseopersicina*; *Syncy.*, *Synechocystis* PCC 6803; *Synco.*, *Synechococcus* PCC 7002; *T.t.*, *Thermus thermophilus*.
(TIF)

Figure S4 **Determination of the pH optimum for NADH-mediated reduction of benzyl viologen (BV) by HoxFU.** The highest specific activity (297 Units / mg of protein) was observed at pH 10 and set to 100%.
(TIF)

Figure S5 **Individual data points of the activity measurements (NADH→BV) used for the determination of the Michaelis Menten constant for BV by non linear regression (R^2^ = 0.945).**
(TIF)

Figure S6 **Individual data points of the activity measurements (NADH→BV) used for the determination of the Michaelis Menten constant for NADH by non linear regression (R^2^ = 0.949).**
(TIF)

Figure S7 **Emission spectrum of HoxFU (4.5 µM) excited at 430 nm.** The spectrum is typical for FMN. RFU, relative fluorescence units.
(TIF)

Figure S8 **Excitation spectrum of HoxFU (4.5 µM). The fluorescence at 525 nm was monitored as a function of the excitation wavelength.** The spectrum is typical for FMN. RFU, relative fluorescence units.
(TIF)

Figure S9 **Cyclic voltammograms for a pyrolytic graphite ‘edge’ electrode modified with 2 µM FMN (black) and** ***R. eutropha*** **HoxFU (grey).** These were recorded at 100 mV/s in a solution of 50 mM Tris-HCl pH 8.0 buffer at 1°C.
(TIF)

Figure S10 **Set of electrochemical experiments designed to confirm that HoxFU remains active in the presence of O_2_, using an inhibition method established by Armstrong and coworkers [Goldet G, Wait AF, Cracknell JA, Vincent KA, Ludwig M, et al. (2008) Hydrogen production under aerobic conditions by membrane bound hydrogenases from** ***Ralstonia*** **species. J Am Chem Soc 130: 11106–11113].** In all experiments the electrode was poised at −412 mV in 0.14 mM NAD^+^, pH 8.0 Tris-HCl buffer, 50 mM. ADP-ribose (ADPR, Sigma) was used without further purification. Panel A shows the effect of injections of ADP-ribose on NAD^+^ reduction by HoxFU, confirming that ADP-ribose is an inhibitor. Panel B shows O_2_ reduction by an unmodified electrode following injection of O_2_, and confirms that injections of ADP-ribose do not affect the O_2_ reduction current. Panel C shows an analogous experiment on a film of HoxFU, showing that injections of ADP-ribose now cause a drop in current magnitude, confirming that HoxFU must remain active in the presence of O_2_, with electrocatalytic NAD^+^ reduction current contributing to the total negative current.
(TIF)

Table S1 **Purification of HoxFU from** ***R. eutropha*** **.**
(TIF)

Table S2 **Results from peptide mass fingerprinting analyses of purified HoxFU protein.**
(TIF)
