# Supplementary figures and images for: Catalytic Properties of the Isolated Diaphorase Fragment of the NAD+-Reducing [NiFe]-Hydrogenase from Ralstonia eutropha
Source: PLoS One. 2011 Oct 10;6(10):e25939. doi: 10.1371/journal.pone.0025939 (PMC3189943; doi:10.1371/journal.pone.0025939)

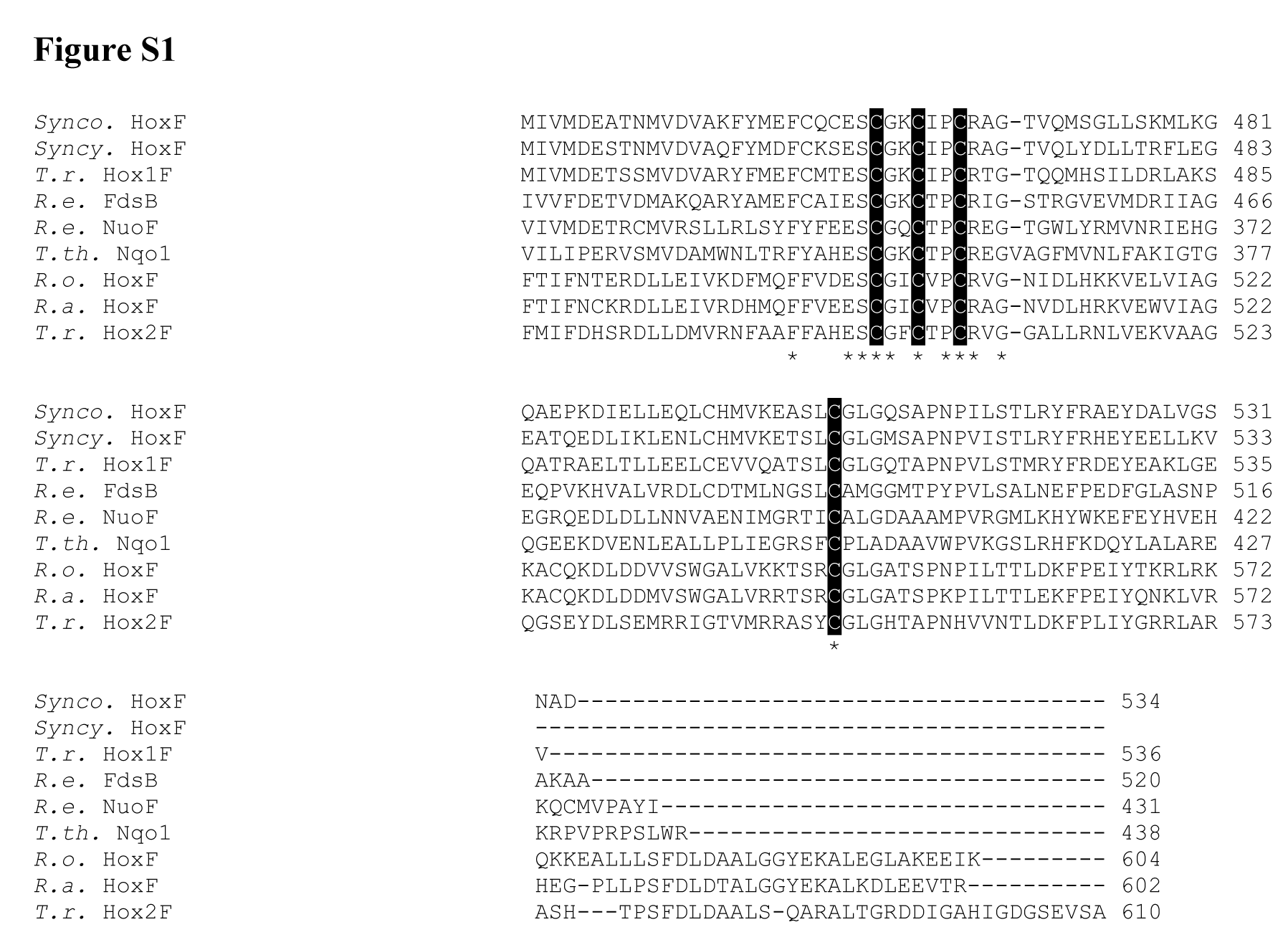

Supplement: Figure S1 — Sequence alignment of the C- terminal part of HoxF from the R. eutropha SH with subunits from related hydrogenases, the FdsB subunit of the NAD+-dependent formate dehydrogenase of R. eutropha and the Complex I subunits NuoF from R. eutropha and Nqo1 from T. thermophilus . Cysteine residues involved in coordination of the [4Fe4S] cluster in Nqo1 of T. thermophilus [14] which are conserved in all the other proteins are boxed. Abbreviations: R.e., Ralstonia eutropha; R.o., Rhodococcus opacus; T.r., Thiocapsa roseopersicina; Syncy., Synechocystis PCC 6803; Synco., Synechococcus PCC 7002; T.th., Thermus thermophilus. Amino acids residues conserved in all proteins are marked with a *. (TIF) [file pone.0025939.s001.tif]

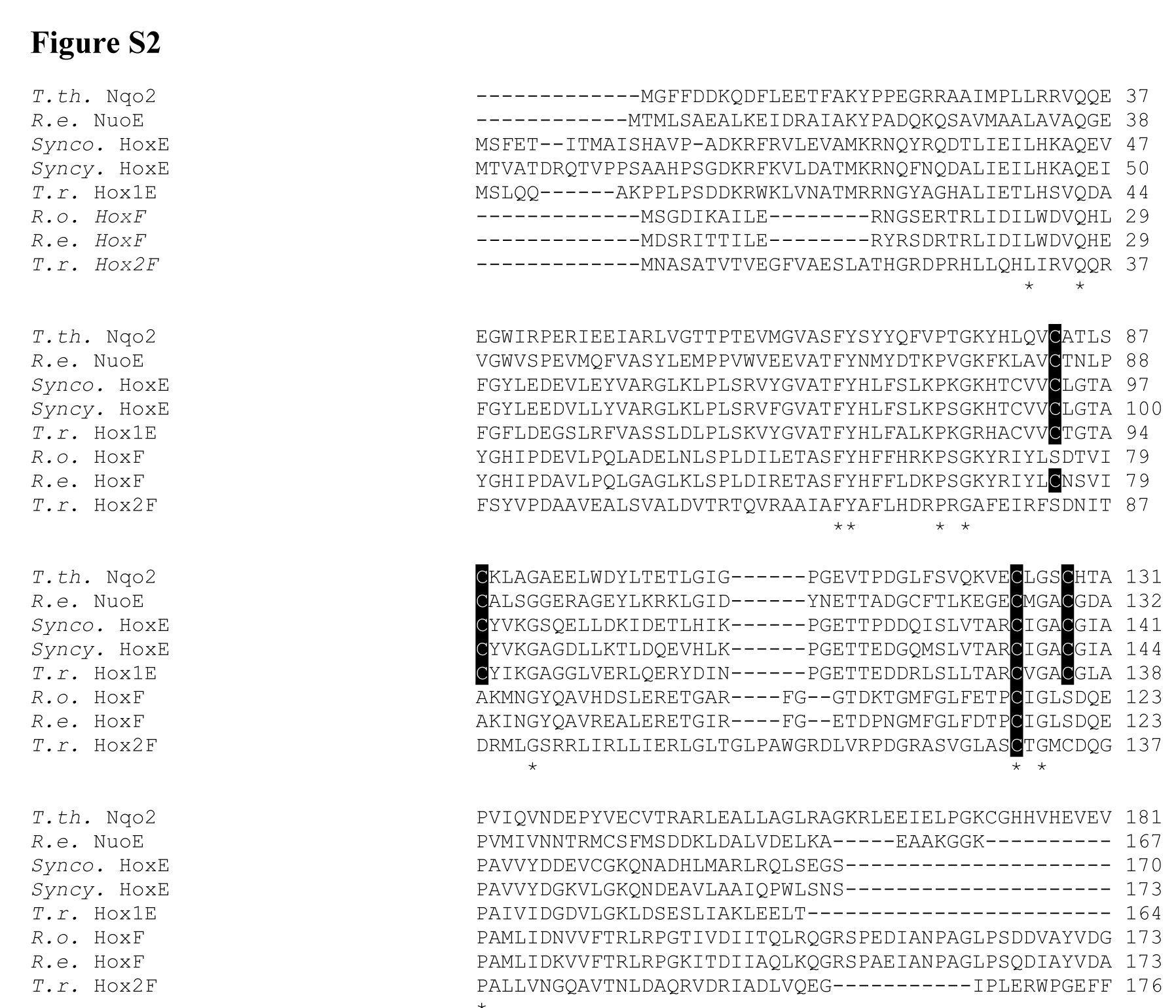

Supplement: Figure S2 — Sequence alignment of the N- terminal part of HoxF from the R. eutropha SH with subunits from related hydrogenases and the Complex I subunits NuoE from R. eutropha and Nqo2 from T. thermophilus . Cysteine residues involved in coordination of the [2Fe2S] cluster in Nqo2 [14] are indicated by black boxes. The absence of most of these cysteine residues suggests that HoxF of R. eutropha does not contain a [2Fe2S] cluster. Abbreviations: R.e., Ralstonia eutropha; R.o., Rhodococcus opacus; T.r., Thiocapsa roseopersicina; Syncy., Synechocystis PCC 6803; Synco., Synechococcus PCC 7002; T.th., Thermus thermophilus. Amino acids residues conserved in all proteins are marked with a *. The N-terminal part of HoxF shares similarities with HoxE from cyanobacterial bidirectional hydrogenases and Nqo2 from complex 1 whereas the C- terminal part of HoxF is homologous to cyanobacterial HoxF proteins and Nqo1 from complex 1. This bipartite nature indicates that of the R. eutropha HoxF represents a fusion protein of two Complex I subunits. (TIF) [file pone.0025939.s002.tif]

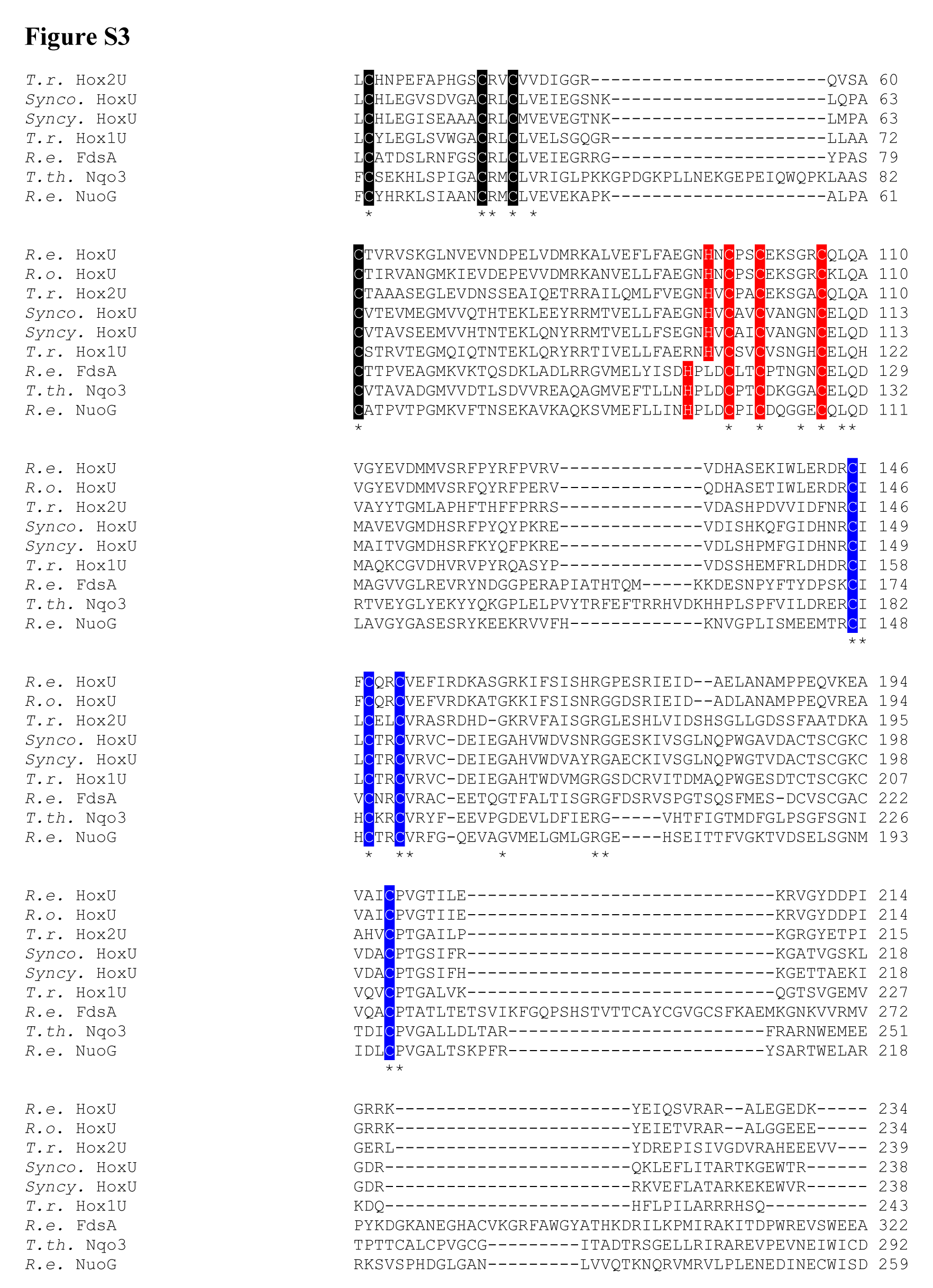

Supplement: Figure S3 — Sequence alignment of the HoxU from the R. eutropha SH with subunits from related hydrogenases, the FdsA subunit of the NAD+-dependent formate dehydrogenase of R. eutropha and the N-terminal parts of the Complex I subunits NuoG from R. eutropha and Nqo3 from T. thermophilus . Cysteine and histidine residues involved in coordination of the [2Fe2S] cluster (black) and the two [4Fe4S] clusters (red+blue) in Nqo3 [14] are highlighted. Amino acids residues conserved in all proteins are marked with a *. Abbreviations: R.e., Ralstonia eutropha; R.o., Rhodococcus opacus; T.r., Thiocapsa roseopersicina; Syncy., Synechocystis PCC 6803; Synco., Synechococcus PCC 7002; T.t., Thermus thermophilus. (TIF) [file pone.0025939.s003.tif]

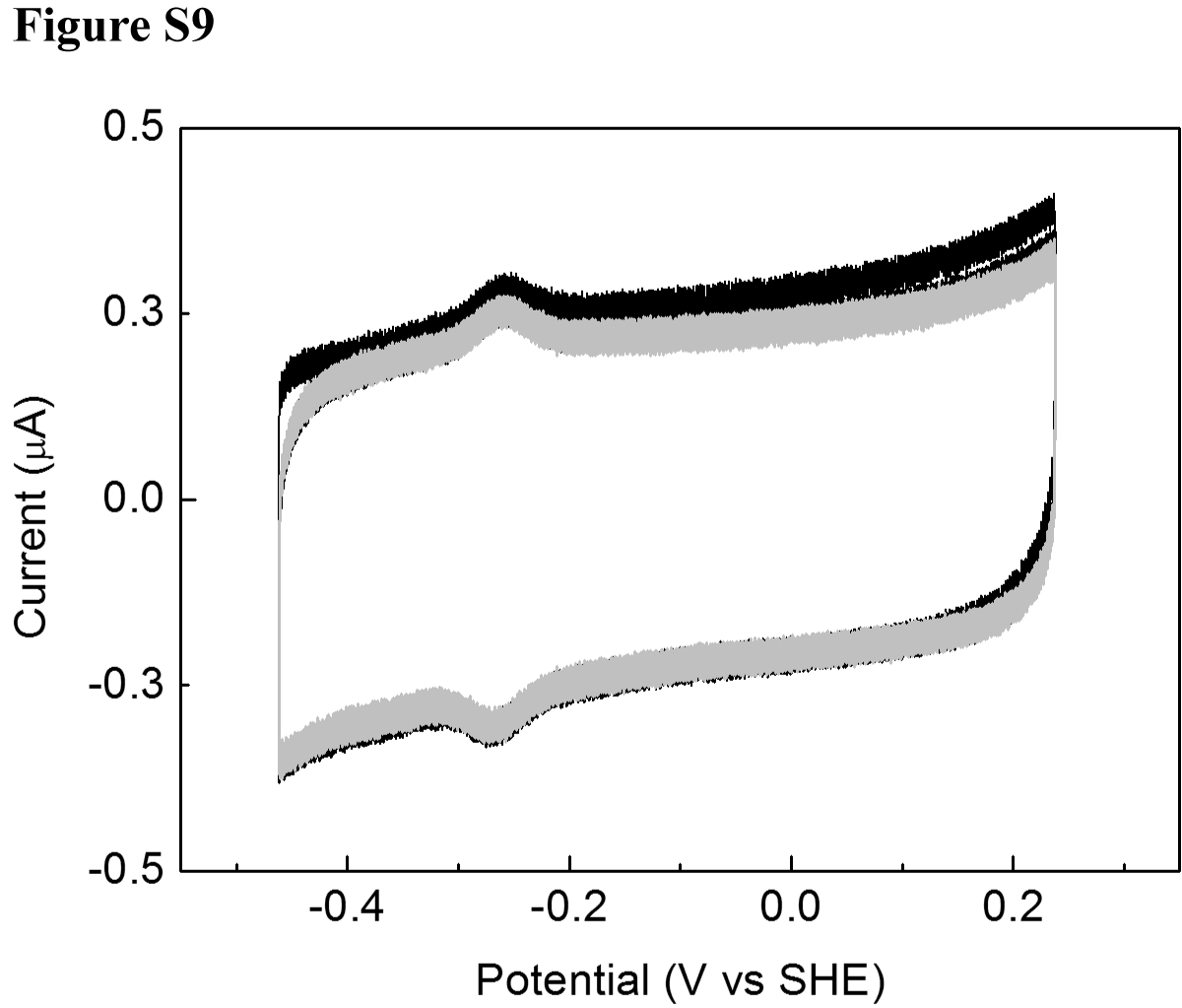

Supplement: Figure S9 — Cyclic voltammograms for a pyrolytic graphite ‘edge’ electrode modified with 2 µM FMN (black) and R. eutropha HoxFU (grey). These were recorded at 100 mV/s in a solution of 50 mM Tris-HCl pH 8.0 buffer at 1°C. (TIF) [file pone.0025939.s009.tif]

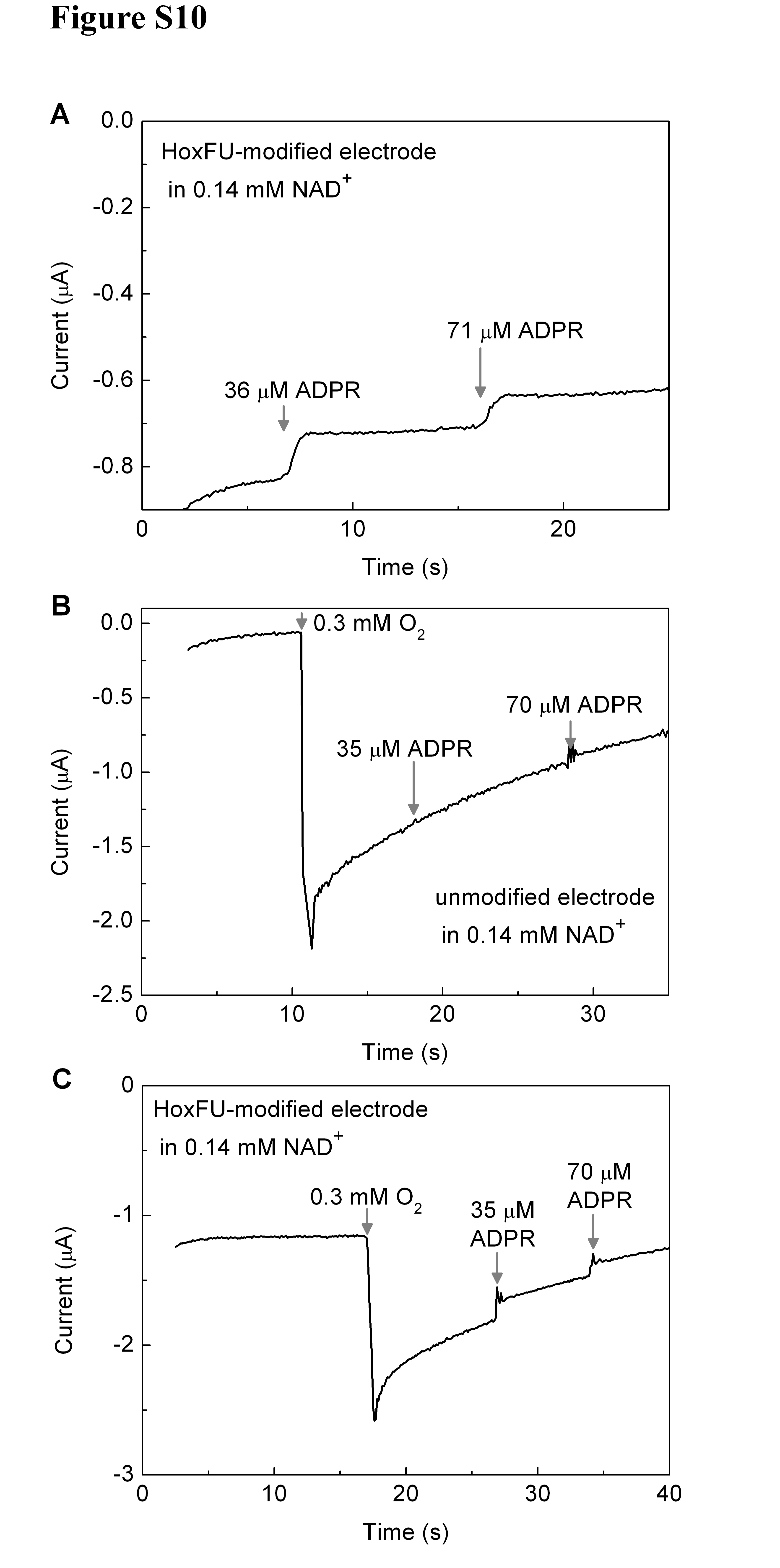

Supplement: Figure S10 — Set of electrochemical experiments designed to confirm that HoxFU remains active in the presence of O2, using an inhibition method established by Armstrong and coworkers [Goldet G, Wait AF, Cracknell JA, Vincent KA, Ludwig M, et al. (2008) Hydrogen production under aerobic conditions by membrane bound hydrogenases from Ralstonia species. J Am Chem Soc 130: 11106–11113]. In all experiments the electrode was poised at −412 mV in 0.14 mM NAD+, pH 8.0 Tris-HCl buffer, 50 mM. ADP-ribose (ADPR, Sigma) was used without further purification. Panel A shows the effect of injections of ADP-ribose on NAD+ reduction by HoxFU, confirming that ADP-ribose is an inhibitor. Panel B shows O2 reduction by an unmodified electrode following injection of O2, and confirms that injections of ADP-ribose do not affect the O2 reduction current. Panel C shows an analogous experiment on a film of HoxFU, showing that injections of ADP-ribose now cause a drop in current magnitude, confirming that HoxFU must remain active in the presence of O2, with electrocatalytic NAD+ reduction current contributing to the total negative current. (TIF) [file pone.0025939.s010.tif]

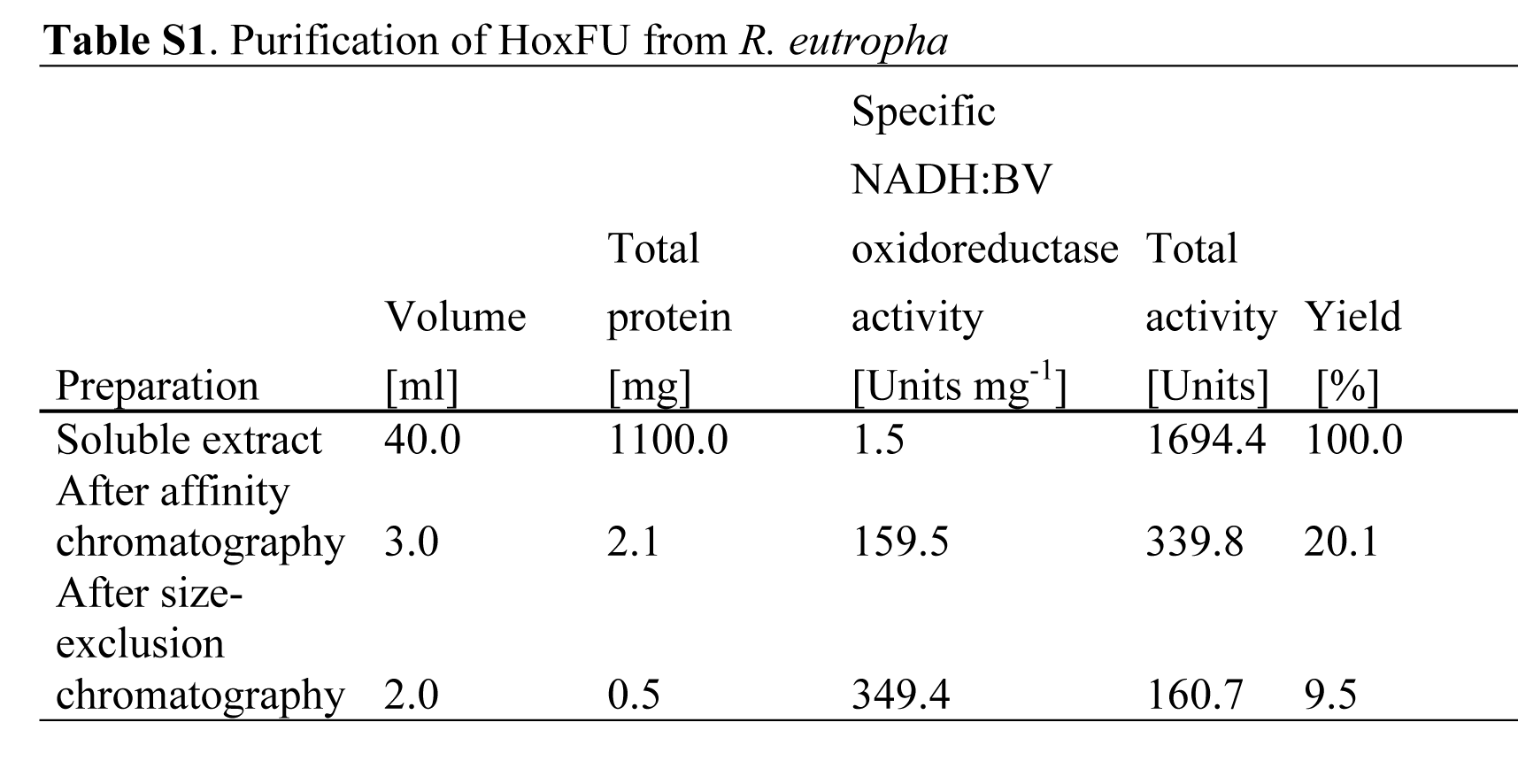

Supplement: Table S1 — Purification of HoxFU from R. eutropha . (TIF) [file pone.0025939.s011.tif]

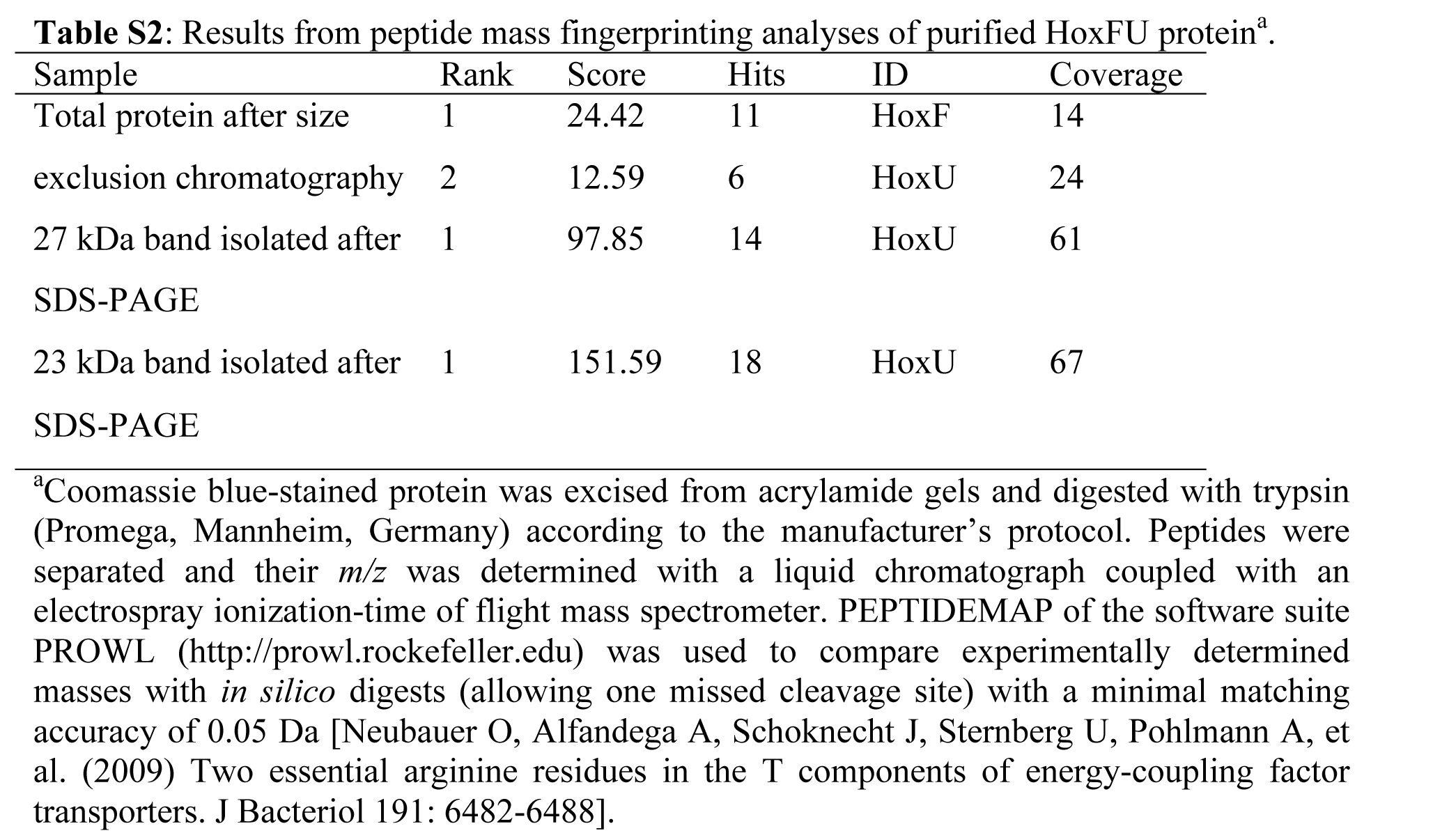

Supplement: Table S2 — Results from peptide mass fingerprinting analyses of purified HoxFU protein. (TIF) [file pone.0025939.s012.tif]
